# What Molecular Recognition Systems Do Mesenchymal Stem Cells/Medicinal Signaling Cells (MSC) Use to Facilitate Cell-Cell and Cell Matrix Interactions? A Review of Evidence and Options

**DOI:** 10.3390/ijms22168637

**Published:** 2021-08-11

**Authors:** David A. Hart

**Affiliations:** 1 Department of Surgery and Faculty of Kinesiology, University of Calgary, Calgary, AB T2N 4N1, Canada; hartd@ucalgary.ca; 2 McCaig Institute for Bone & Joint Health, University of Calgary, Calgary, AB T2N 4N1, Canada; 3 Alberta Health Services Bone & Joint Health Strategic Clinical Network, Edmonton, AB T5H 3E4, Canada; 4 Centre for Hip Health & Mobility, University of British Columbia, Vancouver, BC V5Z 1M9, Canada

**Keywords:** mesenchymal stem cells, medicinal signaling cells, cell surface, recognition systems, cell-cell interactions, cell-matrix interactions

## Abstract

Mesenchymal stem cells, also called medicinal signaling cells (MSC), have been studied regarding their potential to facilitate tissue repair for >30 years. Such cells, derived from multiple tissues and species, are capable of differentiation to a number of lineages (chondrocytes, adipocytes, bone cells). However, MSC are believed to be quite heterogeneous with regard to several characteristics, and the large number of studies performed thus far have met with limited or restricted success. Thus, there is more to understand about these cells, including the molecular recognition systems that are used by these cells to perform their functions, to enhance the realization of their potential to effect tissue repair. This perspective article reviews what is known regarding the recognition systems available to MSC, the possible systems that could be looked for, and alternatives to enhance their localization to specific injury sites and increase their subsequent facilitation of tissue repair. MSC are reported to express recognition molecules of the integrin family. However, there are a number of other recognition molecules that also could be involved such as lectins, inducible lectins, or even a MSC-specific family of molecules unique to these cells. Finally, it may be possible to engineer expression of recognition molecules on the surface of MSC to enhance their function in vivo artificially. Thus, improved understanding of recognition molecules on MSC could further their success in fostering tissue repair.

## 1. Background

Over the past >30 years, the potential of adult-derived mesenchymal stem cells (MSC) to effect repair or regeneration of damaged tissues of the musculoskeletal system has been investigated by a myriad of researchers. First described by Caplan’s group, MSC were defined by a set of criteria based on adherence to plastic in vitro, expression of some cell surface molecules and not others, and ability to be induced to differentiate into several lineages (reviewed in [1,2,3]). Since that time, successes regarding their use to regenerate damaged tissues have been limited and most attempts have not yet lived up to the initial hype as to their potential, but some advances have been made in areas such as tissue engineering for cartilage repair [4,5]. Evaluation of MSC from multiple tissue sources (i.e., bone marrow, adipose tissue, synovium, placenta, Wharton’s jelly, synovial fluid, to name a few), and applications of free MSC, MSC in artificial scaffolds, and MSC in scaffold-free constructs has yielded much useful information, but clinical success has still been limited thus far.

This limited success has prompted Caplan to suggest that perhaps the term mesenchymal stem cells is a misnomer, and these cells should be called medicinal signaling cells to reflect the fact that in vivo they often appear to be pericytes and function to secrete trophic factors [6] and shed exosomes (discussed in [7]), and thus the primary function of the cells may be in more of a paracrine role [8]. It is yet to be proven that this “default” position is the only function of MSC, as it is possible that there are multiple subsets of MSC [9]. Furthermore, even if the primary function in vivo is as Caplan has recently postulated [8], the successes obtained thus far with MSC as stem/progenitor cells would indicate that these cells do have a potential that can be exploited if treated in a specific manner [3,4,10,11]. In addition, MSC derived from different tissue sources appear to exhibit preferences to differentiate towards specific lineages in vitro [12,13], possibly indicating that there is some in vivo imprinted information in these cells, possibly via epigenetic signatures (discussed in [14]). For lineages such as the osteogenic lineage, additional evidence indicates that long non-coding RNAs and microRNAs play key roles in such epigenetic regulation of this lineage [15].

Another feature of MSC that can be derived from the literature is that MSC populations obtained from sources such as bone marrow [16,17], adipose tissue [18], synovium [9], or synovial fluid [12] are very heterogeneous (reviewed in [19]). Using limiting dilution analysis to isolate clonal populations, it is clear that such clones are heterogeneous with regard to proliferation rates, doubling potential, lineage preferences, and molecular responses before and after differentiation [12]. In addition, some reports would indicate that such clonal cells are not stable and exhibit heterogeneity even after cloning [20]. However, the latter may be an artifact of the in vitro culturing conditions [21,22].

From this >30 years of research effort, a considerable body of knowledge of what MSC are and what they can do [23,24,25], as well as what they are not, has accumulated, but it is clear that there is much about these cells that remains to be elucidated. One area that would seem to be critical to understanding MSC and their potential is elucidation of the molecular recognition systems they use to facilitate cell-cell and cell-matrix interactions. However, there is a paucity of information available in this regard, and this deficiency likely is in need of research attention to assist in the realization of the potential of MSC to repair and regenerate tissues that have lost their integrity for a variety of reasons. Such undertakings would not be trivial. Some systems may be constitutive or inducible, relate to localization issues or function in specific environments, be simple or complex regarding the number of components, may be of high or low affinity or avidity, may recognize protein (linear or conformational determinates) or carbohydrate moieties, and may be highly specific for a molecule or specific for classes of ligands. Finally, for those MSC that require proteinase treatment to be released from their tissue/organ, that could modify cell surface expression or be a modifying signal in of itself, particularly if they are subsequently cultured on plastic in the presence of a protein source such as fetal calf serum.

Studies by Liu et al. [26] have characterized expression of receptors on MSC including ICAM-1, Gal-9, PD-L1, TIGT, CD200, and CXCR4. Zha et al. [27] have reported the expression of NGF receptors (TrkA and p75NTR) on MSCs and discussed their potential role in MSC functions. Therefore, MSC often express specific receptors for responsiveness to specific mediators, but whether they express additional classes of receptors that define their roles and activities remains to be delineated.

Towards that goal, a recent report by Matta et al. [28] examined the cell surface “surfaceome” of a MSC population before and after induction of differentiation using a proteomic approach. They reported the presence of a large number of molecules from several classes or with many functions. They did detect HLA Class I molecules, as they should, but did not detect HLA Class II molecules. Furthermore, a number of members of receptor families were detected including representatives of the integrin family, other members of the immunoglobulin family, as well as several members of the efrin and plexin family. While the intent of the studies was not to identify potential recognition system molecules, the approach utilized demonstrated its potential for further characterization of MSC from different sources and in different states regarding receptors that might serve the above-mentioned roles in MSC.

Therefore, the intent of this review is to discuss the direct and indirect evidence for recognition molecules on the surface of MSC, as well as other possible recognition systems that such cells could use in the performance of their functions. Finally, approaches to enhance the functionality of MSC by artificially engineering surface molecules to enhance their recognition activity is addressed.

## 2. Does MSC Heterogeneity also Imply Their Potential Use of a Cell Surface Recognition System That Reflects a Clone-Specific Heterogeneity?

As discussed above, adult MSC are very heterogeneous with regard to their clonal characteristics, dependent on tissue source, existence of subsets, proliferation characteristics, mechanical environment, and function regarding lineage potential after differentiation (reviewed in [19]). If one assumes such characteristics are not an artifact of their in vitro culture and definition [20,21,22,29], the question then arises as to whether such features are accompanied by a cell-surface recognition system that is equally heterogeneous. Currently, much of how such MSC heterogeneity arises and is maintained still remains to be answered, although some aspects have been discussed [19]. Whether such heterogeneity is also associated with development of a clonally unique cell surface recognition system remains to be addressed.

Interestingly, many of the features we ascribe to MSC are also present in other systems in the body. Thus, the bone marrow (BM) hematopoietic system responsible for red blood cells and cells of the innate immune system (i.e., PMN, monocytes, mast cells) arise via specific lineages of increasing restriction from pluripotent progenitor cells, which in turn arise from stem cells (reviewed in [30,31,32]). The BM stromal cells that have been labeled MSC also appear to support some of the hematopoietic functions in the BM (discussed in [16]). Heterogeneity in cells of the specific immune system can also arise in the BM, as well as in the thymus of mammals.

Cells of the innate immune system arising from the BM can be released and then some, such as monocytes, can further differentiate in specific tissues or in response to signals arising from sites of acute and chronic inflammation, as well as tissue damage. Mast cell precursors can also give rise to tissue mast cells and mucosal mast cells (discussed in [33]). Thus, central and peripheral heterogeneity is apparent and related to function in specific environments. However, the heterogeneity associated with these cells is fairly limited and they appear to function more as a class of cells than a set of heterogeneous individual cells.

For cells of the specific immune system that are lymphocytes, such heterogeneity is accompanied by a diverse recognition system on both the B-lymphocytes (bone marrow-derived lymphocytes destined to make antibodies in a clonal-specific manner) and T-lymphocytes (thymus-derived lymphocytes destined to perform a variety of functions via a unique recognition system and via release of cytokines and other trophic factors). Some of these cells circulate in the blood, but other subpopulations are located in organs such as the spleen, lymph nodes, Peyer’s patches, and in the lung.

Cells of the specific immune system such as lymphocytes have at least three systems that can function as recognition systems. On B-lymphocytes, the unique cell-surface antibody that is clonally expressed has an idiotype that can recognize an antigen (**external recognition**) and can also recognize and be recognized by a cell bearing an anti-idiotype (**internal recognition**) [34,35,36]. This clonally expressed system on lymphocytes thus has a bifunctional function (**external and internal recognition**). The third recognition system of lymphocytes is related to localization (**function recognition**) at sites of infection, tissue damage and inflammation through chemokines and cytokines, and via specific receptors on the cells. Thus, even in this system, individual cells or subsets can have multiple recognition systems at play regarding function and regulations.

Thus far, there is no evidence for an analogous system to that expressed on lymphocytes also occurring on MSC. Furthermore, if it is a correct assumption that the primary function of MSC relates to their localization in tissues as pericytes, there may not be a good rationale for why MSC would need such a specific recognition system. However, further studies are needed to support or refute such a system on MSC, and the heterogeneity in other characteristics may reflect some other purpose (or even be at least partially an artifact of culturing) [20,21,22].

## 3. What Other Cell Surface Recognition Systems Could MSC Use, Either as Pericytes in Tissues or as Circulating Cells?

Whether MSC express receptors analogous to those on lymphocytes is only one issue as MSC may also express other receptors that participate in other functions. These include molecules of the classifications detailed below and summarized in Table 1.

The cell surface of cells contains a number of sugars in varying configurations and amounts attached to proteins (glycoproteins) and lipids (i.e., gangliosides, other glycolipids) (reviewed in [37]). The configurations and length of the carbohydrate chains are determined by specific enzymes (glycosyl transferases) that contribute to transferring sugars in unique “patterns” to molecules destined for the cell surface. Conversely, while on the surface of cells, the pattern of glycosylation can also be modified by exogenous glycosidases. Thus, some authors say that carbohydrates are the “third alphabet of life” [37] with their own complexities. Interestingly, using a panel of fluorescently labeled plant lectins, Talaei-Khozanti et al. [38] reported that MSC derived from different sources (bone marrow, adipose tissue, cord blood, Wharton’s jelly) exhibited different patterns of binding of such plant lectins with defined sugar specificities. The purpose of such variation is not known presently, but it could serve specific functions related to cell-cell recognition, localization, and cell-matrix interactions.

Molecules that recognize unique carbohydrates can also serve as recognition systems in mammals and microbes and are thus diverse and likely evolutionarily old. Certainly, antibodies that recognize elements of the ABO blood system are one example, as well as other antibodies targeting carbohydrates (reviewed in [39]). In addition, lectins on the surface of cells can recognize specific simple or complex carbohydrate structures (reviewed in [40,41]). As tissue-specific expression of different glycosyl transferases could impart specificity to the ECM of a tissue, such a recognition system would have some potential to provide a unique recognition system for MSC. It is already known that some molecules that have undergone modification of their glycosyl elements are cleared from the circulation by lectins on the surface of cells of the liver (reviewed in [42]). In addition, cell surface molecules such as gangliosides (glycosphingolipids) can also participate in relevant cell activities (i.e., cell-cell recognition, adhesion) in stem cells (discussed in [43]). Gangliosides can be recognized by cell-associated lectins (reviewed in [41]), and glycans on surface molecules are dynamic and can be influenced by environmental signals (reviewed in [44]).

Interestingly, most bacterial toxins are lectins and use specific carbohydrate configurations as recognition units in order to bind and infect cells [45,46,47]. Variation in glycosyl transferase systems by different species can determine whether a species is sensitive or insensitive to a toxin such as the diphtheria toxin (reviewed in [46]). Other mammalian lectins (i.e., galectin-3 and others) are also used to facilitate communication between cells [40,48]. Thus, carbohydrate moieties and their cognate lectins constitute a recognition system that is also widely used in nature, but is one less stringent than those on B- and T-lymphocytes.

In addition to classical lectins, with the C-type lectins a major family [49], other carbohydrate-recognizing molecules such as Siglecs and selectins may also serve specific recognition functions [39]. Selectins are surface molecules that facilitate the localization of lymphocytes to specific altered environments such as sites of inflammation. Thus, lymphocytes also express elements of recognition systems required for functioning in special environments. Siglecs are lectin-like molecules belonging to the immunoglobulin family that are also expressed on cells of the immune system [39,50]. The Siglecs are a family of molecules that are considered I-type lectins. Whether MSC from different tissues express members of the Siglec family remains to be determined.

Another recognition system that is evident in multiple systems is that of the integrin family of molecules that recognize specific peptide sequences such as the RGD sequence in fibronectin or other sequences on ECM molecules such as collagen, laminin, and others (reviewed in [51,52,53,54]). Through a combination of different protein chains of family members, a variety of recognition specificities can be achieved. When expressed on different cell types, the integrin system can recognize different classes of molecules in a more general manner. In addition, from the work of Matta et al. [28] some evidence for the expression of members of the integrin family on the surface of MSC has been elucidated. However, integrins on the surface of cells may be present in an inactive form that requires activation to be functional [55,56].

An issue that should be mentioned regarding the above-mentioned recognition systems is that of affinity and avidity of the receptor for the molecule(s) in question. For instance, an IgG antibody can be of high affinity and with two identical binding sites bind tightly to the molecule being recognized. In contrast, an IgM antibody with 10 identical binding sites may bind with higher avidity to the target molecule even if each site is of a lower affinity, a thermodynamic property of the molecule. Similarly, many carbohydrate recognizing systems are of lower affinity than corresponding protein/peptide-recognizing systems. This concept of affinity/avidity and target chemistry may be relevant to any recognition system that MSC may use. Relevant to this point is the recent report from Taichi et al. [57] discussing the importance of epitope density and stability of recognition.

With regard to MSC, some are localized in specific tissues, while others appear to circulate in the blood or be residents in the synovial fluid as free cells. Such cells appear to be heterogeneous at multiple levels. For instance, cells defined as MSC found in synovial fluid are very heterogeneous with regard to proliferation, doubling life, lineage specificity, and molecular response to stimuli [9,12,16,17]. How such heterogeneity arises, its relation to potential molecular recognition systems, and association with specific functions remains largely unknown at the present time.

## 4. Do Different Subsets of MSC Use Different Recognition Systems and Are They Constitutive or Inducible?

The answer to this question is currently mainly unknown. However, there are a few insights that may help shape thinking of what options there may be. First, injection of millions of bone marrow-derived MSC into the knee of an injured/damaged preclinical model leads to the retention of only a very small number of cells after a day or so (discussed in [58,59,60]), and there may be some species differences in localization frequency (discussed in [58]), a finding that may complicate generalizations from data obtained with cells from a single species. This consistent finding would indicate that whatever constitutive recognition system is involved, it is potentially one with low affinity or avidity, is highly specific for a defined target, or it is an inducible system that remains “hidden” functionally until the appropriate inducer is present. However, there may also be trivial explanations that are related to the possibility that culturing the MSC in vitro to expand the population has led to the de-differentiation of the cells with an accompanying loss of the expression of the molecules of the recognition system [20]. Alternatively, perhaps treating adherent cells with a proteinase to release them from plastic may have removed components of a trypsin-sensitive protein recognition system that does not re-express readily.

Assuming there is no trivial explanation for the results obtained thus far, then there are a number of possibilities for what MSC could use for molecular recognition systems as discussed above. It should be noted that the options discussed are not mutually exclusive, and the MSC could use >1 system for specific purposes. However, the properties of any recognition system or system must account for the relative inability to find significant numbers of systemically-injected MSC to localize to sites of tissue damage or stay at sites of tissue damage when injected locally.

It should also be kept in mind that tissue-localized MSC may represent subpopulations of MSC that have undergone epigenetic modifications to become tissue-specific and, thus, do not need a special recognition system(s) when in the context of the microvasculature, parenchymal cells, and ECM of the tissue. However, such MSC that are pericytes are still adherent to the cells and matrix that they are in contact with in that tissue. Thus, one would suspect that tissue-specific MSC would preferentially bind to ECM or endothelial cells (EC) from the tissue of origin rather than ECM and EC from other tissues/organs. This possibility could be assessed by investigating the binding of a population of MSC to the ECM of different tissues. Equine MSC are reported to secrete members of the galectin family of lectins [61], and galectin-1 is reported to modify the motility of human umbilical cord MSC [62], so there is some precedence for these lectins to be involved with MSC.

Thus, the BM and circulating MSC may have a cell surface recognition system that recognizes carbohydrates with a pattern of cell surface lectins that allows for some tissue affinity, but the pattern may be of low avidity. Thus, tissue-associated MSC could use such a system to form stable relationships with tissue ECM and cells of the microvasculature. This possibility could be further tested with oligosaccharides comprised of specific monosaccharides of defined structure.

An alternative concept is that MSC contain class-specific carbohydrate configurations on glycoproteins and glycolipids that are targeted by lectins in the various tissues/organs. Thus, they do not recognize targets but are recognized by the cognate receptors of low avidity dispersed in intact and damaged tissues. This type of carbohydrate recognition may have some limitations due to the activity of glycosidases that could disrupt the integrity of the system.

Another potential possibility is that MSC may use different systems with regard to localization activities and those of their function in the context of a specific localization. One example of this was reported with synovial fluid MSC [63,64], a cell population that can be obtained without any pretreatment. The authors reported that synovial fluid MSC obtained from normal knee joints would aggregate spontaneously when treated with a cocktail of factors designed to induce chondrogenesis [63]. However, MSC derived from the synovial fluid of patients with knee OA would not and required culture in micromass conditions to form aggregates. Thus, normal SF MSC can be induced to express a self-recognition molecule in response to the differentiation cocktail, but analogous cells from knees of patients with OA cannot. Interestingly, normal MSC exposed to the cytokine MCP-1, a molecule that binds to the CXCL-2 receptor on monocytes and other cells, leads to the normal cells becoming unable to self-recognize and form cell aggregates [64]. The concentration of MCP-1 is elevated in SF from patients with knee OA (discussed in [64]). Therefore, MCP-1 may be important in the functioning of the MSC in this environment. Whether the recognition system in question is unique to MSC derived from the SF is unknown, but this question could be answered in part by mixing normal SF MSC with labeled MSC from other compartments such as BM or blood.

While further observations with SF MSC regarding MCP-1 and self-aggregating phenomena have not been reported, the observations themselves can provide some valuable clues as to how the process is regulated and the characterization of the type of cell surface recognition molecules that are involved. Thus, there are several interesting points that can be gleaned from the above-described studies by Krawetz et al. [63] and Harris et al. [64]. The first relates to the early events regarding the aggregation of MSC that form naturally in response to chondrogenic differentiation signals. That is, does aggregation initiate around a unique MSC subtype in the population, or does it occur with involvement of all of the MSC in the population via a specific recognition system or stochastically? Since the MSC were in a 2D culture system on a plastic dish, there must have been some molecular stimulation to move and form a stable aggregate. To gain a better understanding of the phenomenon of the aggregation of normal SF MSC in differentiating medium, one could use time-lapse photography of cell cultures, attempt to inhibit aggregation with simple sugars, or assess cell surface molecule changes after exposure to a differentiation medium using the methodology of Matta et al. [28] or perhaps lectin arrays [65,66].

From some perspectives, the aggregation of normal synovial fluid MSC in the presence of the chondrogenic differentiation medium resembles that of the aggregation of the slime mold Dictyostelium in response to stress. Thus, when stressed the single celled organisms migrate towards a cAMP gradient to form a stable aggregate that then differentiates further into a fruiting body (reviewed in [67]). The aggregates may use a primitive carbohydrate-lectin system for stabilization [68]. Interestingly, this aggregation appears to involve elements of the intracellular GSK-3 system [69]. Of further interest is that MCP-1 involvement with cells also involves the GSK-3 system [70]. Thus, there may be some parallels between the recognition systems used by MSC and Dictyostelium to accomplish development of a stable aggregate.

Secondly, is the molecule responsible for the self-aggregating activity of SF MSC induced by the combination of components in the chondrocyte differentiating medium (containing dexamethasone, BMP-2, insulin) or one specific component? The answer to this question could be readily pursued by exposing a population of SF MSC to the individual components of the differentiating medium and observing the aggregation activity.

Thirdly, at least a subset of MSC in the normal SF must express receptors for MCP-1 (CCR2), but whether it is the bulk of the cells or some unique subset is not evident presently. The answer to this question could be readily approached with fluorescent MCP-1 and assessment via immunolocalization techniques or FACS analysis.

Thus, there is the possibility that in the differentiation media the MSC are induced to express a common receptor for a molecule such as a common glycan moiety on the cell surface. Furthermore, it would be possible to use MSC from different tissue sources, such as those used by Talaei-Khozani et al. [38], to determine whether the findings with normal SF MSC can be generalized with MSC from other sources and in response to differentiation medium directed towards other lineages.

Interestingly, exposure of rat MSC to lithium, valproate, or lithium + valproate leads to enhanced localization of the cells at the site of a stroke with improved outcomes, possibly due to enhanced expression of the chemokine receptor CXC4 by the treatment [71]. Lithium has been used for several decades in the treatment of bipolar disease and is known to also affect GSK-3beta metabolism and that of the WNT/catenin system (discussed in [72]). Thus, while an indirect link, it would appear that reagents that affect GSK-3 influencing characteristics of at least some MSC is consistent with alterations in their molecular recognition systems. This link to GSK-3beta influencing MSC recognition systems is further supported by reports that MCP-1 can influence GSK-3beta in cells [66] and thus may also relate to the studies discussed above.

However, it should be noted that in the studies by Tsai et al. [71], the increases in cell binding were significantly increased by exposure to lithium salts, but the number of cells binding to the target area was still very modest. Thus, only a subset of MSC may have been impacted by the exposure to lithium salts. Therefore, this approach should be further explored to investigate both the mechanisms involved and whether only a subset of the cells was affected to enhance localization in that model.

## 5. Do Exosomes from MSC Use a Specific Cell-Recognition System to Facilitate Attachment and Uptake by Cells?

Exosomes (also known as extracellular vesicles) appear to be released from MSC by “blebbing” or shedding from the MSC and having a part of the plasma membrane covering content of several relevant molecules (reviewed in [73,74,75,76]). The content can contain growth factors, microRNA (miRNA), and other bioactive molecules. It is believed that such extracellular vesicles/exosomes can then be taken up by other cells and transfer their contents to the recipient cells to influence their metabolism or behavior (reviewed in [77,78]).

It is likely that exosome generation may not be a random event regarding both the plasma membrane components used and their content, but this remains to be detailed. However, it is known that exosomes can express some of the surface molecules used to identify MSC [79]. As the content of the exosomes can be influenced by cellular conditions [80], there is likely some specificity to the mechanism of formation and release of exosomes from the MSC. Furthermore, it is also likely that the actual molecular content of the membranes is not random, containing some membrane and transmembrane molecules necessary to maintain integrity of the exosomes and to facilitate their recognition and uptake by other cells in vitro and in vivo. Thus far, there is a paucity of evidence regarding how this process occurs and is regulated. However, using exosomes from mast cells leads to functional uptake by MSC via cell surface TGF-beta1 [81], so it may be possible that targeting of exosomes could use similar strategies involving other growth factors and their cognate receptors.

In vivo, MSC in their location as pericytes may generate exosomes that could exhibit a subset of MSC-derived surface recognition molecules on their surface that recognize specific targets on other cells in their organ system (i.e., endothelial cells, other functional cells). Alternatively, such exosomes could express specific glycolipids and glycoproteins recognized by recipient cells via cell surface lectins or proteins recognized by cell surface integrins. Functioning in a paracrine manner may override the need for an extensive recognition system, but certainly, a system that could facilitate exosomes traversing the distances from release and recognition, followed by uptake, may offer an advantage of efficiency. In a migrating system of MSC in blood, such a system may depend more on recognition molecules related to a localization system than a specific exosome system, but this remains to be determined. However, as exosomes may be important in post-MSC localization events, such nuances will be important to elucidate.

It should be noted that release of exosomes is not restricted to MSC and that other cells such as tumor cells can also release exosomes that may play a role in disease onset [82]. Thus, exosomes can likely contribute to both health and disease depending on the circumstance of their release.

## 6. Do MSC and iPSC Use Different or Similar Molecular Recognition Systems?

While the above discussions have focused on potential systems used by MSC and exosomes derived from MSC for recognition of other cells or extracellular matrix, in recent years many investigators have turned to using induced pluripotent stem cells obtained by dedifferentiating mature somatic cells. Thus, by using a cocktail of specific reagents, somatic cells such as a skin fibroblast can be induced to “revert” and become a pluripotent stem cell with characteristics indicating they could be used in the treatment of damaged or disease tissues [83,84]. Recently. Such iPSC have been investigated for the potential treatment of heart diseases (discussed in [85]), neurodegenerative conditions (discussed in [86,87,88,89]), and musculoskeletal conditions such as intervertebral disc repair (reviewed in [90]).

The molecular recognition systems used by iPSC to both localize to specific locations and to integrate functionally are also largely unknown. Therefore, it would be of interest to delineate the systems involved, and at some levels, the identification may be easier than with MSC since one would know what is on the surface of the cells before and after treatment to induce dedifferentiation to become stem-like cells, and one could compare the profiles of fibroblasts from different tissues or cells from similar tissues but derived from different genetic origins during development, i.e., dorsal vs. ventral skin fibroblasts (reviewed in [91,92,93]). Such characterization would potentially enhance the efficacy of the iPSC for various clinical applications, as well as enhance the basic scientific understanding of the molecules used by naturally occurring “stem/progenitor” cells versus those arising on cells dedifferentiated by specific protocols. Such investigations could provide significant impact on how to further advance the use of iPSC in tissue repair and regeneration. In addition, a comparison of the recognition systems used by naturally occurring MSC arising in different compartments, and during aging, with those expressed by corresponding iPSC would also be of scientific interest regarding the breadth of such recognition systems for tissue regeneration in different circumstances. In addition, as iPSC also release extracellular vesicles (exosomes) [94], they could also be compared to the analogous vesicles from MSC.

While the potential cell surface recognition systems used by iPSC are not known, it is difficult to imagine how they might generate a system on their surface analogous to that used by cells of the specific immune system. It is likely that a more traditional system (i.e., lectin-glycan, integrin-matrix molecule) and perhaps one more influenced by developmental considerations would be used by such cells. In addition, they may also be influenced and perhaps somewhat restricted by the somatic cells used for their development as iPSC, but this is speculation at this point.

Therefore, it will be important to compare and contrast the cell surface recognition systems used by MSC and iPSC and how any differences may impact the potential applications of the cells for repair and regeneration of damaged or injured tissues. Furthermore, while not directly linked to recognition systems, it would also be of interest to learn more about changes to the glycomes on the surface of iPSC as they develop “stemness” and comparisons with those on MSC as such changes may relate to recognition by other cells in potential targets in tissues. One might expect that generation of iPSCs may lead to a glycome more resembling that of cells during development, but one that could also be influenced by the history of the somatic cell they are derived from. Interestingly, Hirabayashi et al. [95] reported the existence of a cell surface glycan marker on pluripotent stem cells using a modified lectin probe. As lectin arrays have been developed and used [65,66], this can be done with established methodology.

## 7. Summary

While MSC have been used in a large number of publications, and a myriad number of clinical trials, there is much that is still unknown about these cells that has hindered progress to the goal of exploiting their potential. One of the critical areas that still requires considerable investigation relates to improved understanding of the molecular recognition systems MSC and their subpopulations use to perform their functions, including stable localization of free cells, as well as functions within specific environments in response to local mediators and trophic molecules. This information will help meet expectations and provide insights into what is needed to overcome challenges posed by inflammatory environments such as in OA and inflammatory arthritis [96] as examples. A number of options are available to investigate some of the potential molecular recognition systems (receptors and ligands) to such cells, and it just remains that a focused research effort is applied to elucidate which ones are involved and how they are regulated. Based on current evidence, it is likely that cell surface lectins and their cognate glycans comprise at least one such recognition system. In addition, the glycome on the cell surface of MSC and related cells also may contribute to the recognition of MSC subsets by lectins in different tissues and locations. Some evidence regarding the expression of integrins on the surface of MSC [28] may also indicate that members of this family of recognition molecules may also play a role in MSC recognition systems, but more detail is needed to confirm this conclusion.

It should also be noted that some of the options for recognition systems discussed above for MSC and related exosomes may also hold for induced pluripotent stem/progenitor cells (iPSC), and therefore, lessons learned for MSC may also assist in the successful future application of iPSC and vice-versa. Such understanding would enhance basic understanding of the recognition systems used, as well as enhance their potential for clinical applications.

Finally, until further information and understanding regarding what recognition systems are used by MSC regarding their in vivo functions as pericytes in tissues or as free circulating cells, or the functions researchers ask them to perform in tissue engineering scenarios or regarding localization to areas of damaged tissues, their use for some applications may not meet expectations. However, one approach to circumvent this limitation could potentially enhance specific applications for MSC. That is, to engineer recognition system receptors into MSC so as to target them to specific sites [97,98] or to glycoengineer the surface glycans of cells [99,100,101,102] to make the MSC susceptible to endogenous lectins in target tissues. Some reports of such modifications are listed in Table 2. While this approach may be restricted to allogeneic uses of MSC in some cases, it may offer some advantages for defined applications.

While one may debate whether natural MSC are mesenchymal stem cells or medicinal signaling cells, the fact of the matter is that these cells as mesenchymal stem cells do offer the potential for tissue regeneration. The key to unlocking this potential will be to learn more about these cells, including what recognition systems they use in specific scenarios.

## Figures and Tables

**Table 1:** Summary of potential endogenous molecular recognition system options for MSC.

Type	Present on MSC Surface	Active	Inducible
**Protein-Protein**			
*Cell Surface Integrin*	*YES*	*?*	*?*
*Clonally Specific* **^a^**	*NO-Speculative*	*Likely if present*	*?*
**Protein-Carbohydrate**			
*Lectins* **^b^**	*YES*	*YES*	*Likely*
*Tissue-Specific*			
*Carbohydrate*	*YES*	*YES*	*Possibly* **^d^**
*Configurations* **^c^**			

**^a^** Analogous to specific immune system cell-surface molecules (i.e., IgD, T-cell receptors). **^b^** Multiple lectin family members with differing carbohydrate specificities. **^c^** Location-specific patterns of carbohydrate configurations detected by lectin arrays that could be recognized by specific lectins on other cells or associated with ECM. **^d^** Potentially influenced by induction of specific glycosyltransferases and/or glycosidases.

**Table 2:** An alternative approach—engineering recognition molecules into MSC to influence homing and other functions.

Cell Source	Species	Recognition Molecule/System	Reference
BM-MSC	Rat	CXCR4 (CXC-receptor)	Cheng et al. [103]
BM-MSC	Human	E-selectin Ligands	Dykstra et al. [104]
BM-MSC	Swine	P-selectin Ligand-1 Mimetic	Lo et al. [105]
BM-MSC-EV	Mouse	CD47	Wei et al. [106]
UCord-MSC	Human	CCR2	Kuang et al. [107]
Adipose-MSC	Mouse	LSEC targeted peptide	Liao et al. [108]
Adipose-MSC	Rat	P-selectin Binding Peptide	Yan et al. [109]

BM = bone marrow; EV = extracellular vesicles; UCord = umbilical cord.

## Data Availability

Not applicable.

## References

[1] Pittenger M.F., Discher D.E., Peault B.M., Phinney D.G., Hare J.M., Caplan A.I. (2019). Mesenchymal stem cell perspective: Cell biology to clinical progress. NPI Regen. Med..

[2] Lukomska B., Stanazek L., Zuba-Surma E., Legosz P., Sarzynska S., Drela K. (2019). Challenges and controversies in human mesenchymal stem cell therapy. Stem Cell Int..

[3] Brown C., McKee C., Bakshi S., Walker K., Hakman E., Halassy S., Svinarich D., Dodds R., Govind C.K., Chaudhry G.R. (2019). Mesenchymal stem cells: Cell therapy and regeneration potential. J. Tissue Eng. Regen. Med..

[4] Shimomura K., Yasui Y., Koizumi K., Chijimatsu R., Hart D.A., Yonetani Y., Ando W., Nishii T., Kanamoto T., Horibe S. (2018). First-in-human pilot study of implantation of a scaffold-free tissue-engineered construct generated from autologous synovial mesenchymal stem cells for repair of knee chondral lesions. Am. J. Sports Med..

[5] Shimomura K., Hamada H., Hart D.A., Ando W., Nishii T., Trattnig S., Nehrer S., Nakamura N. (2021). Histological analysis of cartilage defects repaired with an autologous human stem cell construct 48 weeks postimplantation structural details not detected by T2 mapping MRI. Cartilage.

[6] Caplan A.I., Dennis J.E. (2006). Mesenchymal stem cells as trophic mediators. J. Cell Biochem..

[7] Cai J., Wu J., Wang J., Li Y., Hu X., Luo S., Xiang D. (2020). Extracellular vesicles derived from different sources of mesenchymal stem cells: Therapeutic effects and translational potential. Cell Biosci..

[8] Caplan A.I. (2017). Mesenchymal stem cells: Time to change the name. Stem Cells Transl. Med..

[9] Affan A., Al-Jezani N., Railton P., Powell J.N., Krawetz R.J. (2019). Multiple mesenchymal progenitor cell subtypes with distinct functional potential are present within the intimal layer of the hip synovium. BMC Musculoskelet. Disord..

[10] Ando W., Tateishi K., Hart D.A., Katakai D., Tanaka Y., Nakata K., Hashimoto J., Fujie H., Shino K., Yoshikawa H. (2007). Cartilage repair using an in vitro generated scaffold-free tissue-engineered construct derived from porcine synovial mesenchymal stem cells. Biomaterials.

[11] Shimomura K., Ando W., Tateishi K., Nansai R., Fujie H., Hart D.A., Kohda H., Kita K., Kanamoto T., Mae T. (2010). The influence of skeletal maturity on allogenic synovial mesenchymal stem cell-based repair of cartilage in a large animal model. Biomaterials.

[12] Ando W., Kutcher J.J., Krawetz R., Sen A., Nakamura N., Frank C.B., Hart D.A. (2014). Clonal analysis of synovial fluid stem cells to characterize and identify stable mesenchymal stromal cell/mesenchymal progenitor cell phenotypes in a porcine model: A cell source with enhanced commitment to the chondrogenic lineage. Cytotherapy.

[13] Wu H., Gordon J.A.R., Whitfield T.W., Tai P.W.L., van Wijnen A.J., Stein J.L., Stein G.S., Lian J.B. (2017). Chromatin dynamics regulates mesenchymal stem cell lineage specification and differentiation to osteogenesis. Biochim. Biophys. Acta Gene Regul. Mech..

[14] Cakouros D., Gronthos S. (2020). Epigenetic regulators of mesenchymal stem/stromal cell lineage determination. Curr. Osteoporos. Rep..

[15] Lanzilloiti C., De Mattei M., Mazziotta C., Taraballi F., Rotondo J.C., Togon M., Martini F. (2021). Long non-coding RNAs and microRNAs interplay in osteogenic differentiation of mesenchymal stem cells. Front. Cell Dev. Biol..

[16] Sivasubramaniyan K., Lehnen D., Ghazanfari R., Sobiesiak M., Harchandan A., Mortha E., Petkova N., Grimm S., Cerbona F., de Zwart P. (2012). Phenotypic and functional heterogeneity of human bone marrow-and amnion-derived MSC subsets. Ann. N. Y. Acad. Sci..

[17] Liu Y., Monoz N., Bunnell B.A., Logan T.M., Ma T. (2015). Density-dependent metabolic heterogeneity in human mesenchymal stem cells. Stem Cells.

[18] Prieto Gonzalez E.A. (2019). Heterogeneity in adipose stem cells. Adv. Exp. Med. Biol..

[19] McLeod C.M., Mauck R.L. (2017). On the origin and impact of mesenchymal stem cell heterogeneity: New insights and emerging tools for single cell analysis. Eur. Cells Mater..

[20] Rennerfeldt D.A., Van Vliet K.J. (2016). Concise review: When colonies are not clones: Evidence and implications of intracolony heterogeneity in mesenchymal stem cells. Stem Cells.

[21] Bentivegna A., Miloso M., Riva G., Foudah D., Butta V., Dalpra L., Tredici G. (2013). DNA methylation changes during in vitro propagation of human mesenchymal cells: Implications for their genomic stability. Stem Cells Int..

[22] Stultz B.G., McGinnis K., Thompson E.E., Lo Surdo J.L., Bauer S.R., Hursh D.A. (2016). Chromosomal stability of mesenchymal stromal cells during in vitro culture. Cytotherapy.

[23] Tanabe S. (2014). Role of mesenchymal stem cells in cell life and their signaling. World J. Stem Cells.

[24] Ayala-Cuellar A.P., Kang J.H., Jeung E.B., Choi K.C. (2019). Roles of mesenchymal stem cells in tissue regeneration and immunomodulation. Biomed. Ther..

[25] Naji A., Eitoku M., Favier B., Deschaseaux F., Rouas-Freiss N., Suganuma M. (2019). Biological functions of mesenchymal stem cells and clinical implications. Cell Mol. Life Sci..

[26] Liu S., Liu F., Zhou Y., Jin B., Sun Q., Guo S. (2020). Immunosuppresive property of MSCs mediated by cell surface receptors. Front. Immunol..

[27] Zha K., Yang Y., Tian G., Sun Z., Yang Z., Li X., Sui X., Liu S., Zhao J., Guo Q. (2021). Nerve growth factor (NGF) and NGF receptors in mesenchymal stem/stromal cells: Impact on potential therapies. Stem Cells Transl. Med..

[28] Matta C., Boocock D.J., Fellows C.R., Miosge N., Dixon J.E., Liddel S., Smith J., Mobasheri A. (2019). Molecular phenotyping of the surfaceome of migratory chondroprogenitors and mesenchymal stem cells using biotinylation, glycocapture and quantitative LC-MS/MS proteomic analysis. Sci. Rep..

[29] Hart D.A. (2014). Is adipocyte differentiation the default lineage for mesenchymal stem/progenitor cells after mechanical loading? A perspective from space flight and model systems. J. Biomed. Sci. Eng..

[30] Canu G., Ruhrberg C. (2021). First blood: The endothelial origins of hematopoietic progenitors. Angiogenesis.

[31] Challen G.A., Goodell M.A. (2020). Clonal hematopoiesis: Mechanisms driving dominance of stem cell clones. Blood.

[32] Bowers E., Singer K. (2021). Obesity-induced inflammation: The impact of the hematopoiesis stem cell niche. JCI Insight.

[33] Piliponsky A.M., Acharya M., Shubin N.J. (2019). Mast cells in viral, bacterial, and fungal infection immunity. Int. J. Mol. Sci..

[34] Kohler H. (2021). The impact of hydridoma technology on the R&D of idiotypic antibodies. Monoclon. Antibodies Immunodiagn. Immunother..

[35] Jerne N.K. (1984). Idiotypic networks and other preconceived ideas. Immunol. Rev..

[36] Hart D.A., Wang A.L., Pawlak L.L., Nisonoff A. (1972). Suppression of idiotypic specificities in adult mice by administration of anti-idiotypic antibody. J. Exp. Med..

[37] Kaltner H., Abad-Rodriguez J., Corfield A.P., Kopitz J., Gabius H.J. (2019). The sugar code: Letters and vocabulary, writers, editors and readers and biosignificance of functional glycan-lectin pairing. Biochem. J..

[38] Talaei-Khozani T., Aleahmad F., Bazrafshan A., Aliabadi E., Vojdani Z. (2019). Lectin profile variation in mesenchymal stem cells derived from different sources. Cell Tissues Organs.

[39] Smith B.A.H., Bertozzi C.R. (2021). The clinical impact of glycobiology: Targeting selectins, Siglecs and mammalian glycans. Nat. Rev. Drug Discov..

[40] Schnaar R.L. (2016). Glycobiology simplified: Diverse roles of glycan recognition in inflammation. J. Leukoc. Biol..

[41] Ledeen R.W., Kopitz J., Abad-Rodriquez J., Gabius H.J. (2018). Glycan chains of gangliosides: Functional ligands for tissue lectins (Siglecs/Galectins). Prog. Mol. Biol. Transl. Sci..

[42] Hu J., Liu J., Yang D., Lu M., Yin J. (2014). Physiolocal roles of asialoglycoprotein receptors (ASGPRS) variants and recent advances in hepatic-targeted deliever of therapeutic molecules via ASGPRs. Protein Pept. Lett..

[43] Ryu J.S., Ko K., Ko K., Kim J.S., Kim S.U., Chang K.T., Choo Y.K. (2017). Roles of gangiosides in the differentiation of mouse pluripotent stem cells to neural stem cells and neural cells (Review). Mol. Med. Rep..

[44] Grouz-Degroote S., Cavdarli S., Uchimura K., Allain F., Delannoy P. (2020). Glycosylation changes in inflammatory diseases. Adv. Protein Chem. Struct. Biol..

[45] Hart D.A. (1980). Lectins in biological systems: Applications to microbiology. Am. J. Clin. Nutr..

[46] Eidels L., Proia R.L., Hart D.A. (1983). Membrane receptors for bacterial toxins. Microbiol. Rev..

[47] Poole J., Day C.J., von Itzstein M., Paton J.C., Jennings M.P. (2018). Glycointeractions in bacterial pathogenesis. Nat. Rev. Microbiol..

[48] Nakahara S., Raz A. (2006). On the role of galectins in signal transduction. Methods Enzymol..

[49] Keller B.G., Rademacher C. (2020). Allostery in C-type lectins. Curr. Opin. Struct. Biol..

[50] Murugesan G., Weigle B., Crocker P.R. (2021). Siglec and anti-Siglec therapies. Curr. Opin. Chem. Biol..

[51] Mezu-Ndubuishi O.J., Maheshari A. (2020). The role of integrins in inflammation and angiogenesis. Pediatr. Res..

[52] Kadry Y.A., Calderwood D.A. (2020). Chapter 22: Structural and signaling functions of integrins. Biochim. Biophys. Acta Biomembr..

[53] Sani S., Messe M., Fuchs Q., Pierrevelcin M., Laquerriere P., Entz-Werle N., Reita D., Etienne-Selloum N., Bruban V., Choulier L. (2021). Biological relevance of RGD-Integrin subtype-specific ligands in cancer. Chembiochem.

[54] Ou Z., Dolmatova E., Lassegue B., Griendling K.K. (2021). B1- and B2-integrins: Central players in regulating vascular permeability and leukocyte recruitment during acute inflammation. Am. J. Physiol. Heart Circ. Physiol..

[55] Mana G., Valdembri D., Serini G. (2020). Conformationally active integrin endocytosis and traffic: Why, where, when and how?. Biochem. Soc. Trans..

[56] Legate K.R., Wickstrom S.A., Fassler R. (2009). Genetic and cell biological analysis of integrin outside—in signaling. Genes Dev..

[57] Taichi M., Kitazume S., Vong K., Imamaki R., Kurbangalieva A., Taniguchi N., Tanaka K. (2015). Cell surface and in vivo interaction of dendrimetric N-glycoclusters. Glycoconj. J..

[58] Williams A.R., Hare J.M. (2011). Mesenchymal stem cells: Biology, patho-physiology, translational findings, and therapeutic implications for cardiac disease. Circ. Res..

[59] Horie M., Sekiya I., Muneta T., Ichinose S., Matsumoto K., Saito H., Murakami T., Kobayashi E. (2009). Intra-articular injected synovial stem cells differentiate into meniscal cells directly and promote meniscal regeneration without mobilization to distant organs in rat massive meniscal defect. Stem Cells.

[60] Henning R.J. (2011). Stem cells in cardiac repair. Future Cardiol..

[61] Reesink H.L., Sutton R.M., Shurer C.R., Peterson R.P., Tan J.S., Su J., Paszek M.J., Nixon A.J. (2017). Galectin-1 and galectin-3 expression in equine mesenchymal stromal cells (MSCs), synovial fibroblasts and chondrocytes, and the effect of inflammation on MSC motility. Stem Cell Res. Ther..

[62] Yun S.P., Lee S.J., Jung Y.H., Han H.J. (2014). Galectin-1 stimulates motility of human umbilical cord blood-derived mesenchymal stem cells by down regulation of smad2/3-dependent collagen 3/5 and upregulation of NF-kB-dependent fibronectin/lammin5 expression. Cell Death Dis..

[63] Krawetz R.J., Wu Y.E., Martin L., Rattner J.B., Matyas J.R., Hart D.A. (2012). Synovial fluid progenitors expressing CD90+ from normal but not osteoarthritic joints undergo chondrogenic differentiation without micro-mass culture. PLoS ONE.

[64] Harris Q., Seto J., O’Brien K., Lee P.S., Kondo C., Heard B.J., Hart D.A., Krawetz R.J. (2013). Monocyte chemotactic protein-1 inhibits chondrogeneis of synovial progenitor cells: An in vitro study. Stem Cells.

[65] Nand A., Singh V., Wang P., Na J., Zhu J. (2014). Glycoprotein profiling of stem cells using lectin microarray based on surface plasmon resonance imaging. Anal. Biochem..

[66] Katoh A., Fugimaru A., Senthikumar R., Preethy S., Abraham S.J.K. (2020). Articular chondrocytes from osteoarthritis knee joints of elderly, in vitro expanded in thermos-reversible gelation polymer (TGP), exhibiting higher UEA-1 expression in lectin microarray. Regen Ther..

[67] Dormann D., Vasiev B., Weijer C.J. (2002). Becoming multicellular by aggregation; the morphogenesis of the social amoebae Dictyostelium discoideum. J. Biol. Phys..

[68] Cano A., Pestana A. (1984). The role of membrane lectins in Dictyostelium discoideum aggregation as ascertained by specific univalent antibodies against discoidin. J. Cell Biochem..

[69] Plyte S.E., O’Donovan E., Woodgett J.R., Harwood A.J. (1999). Glycogen synthase kinase-3 (GSK-3) is regulated during *Dictyostelium* development via the serpentine receptor cAR3. Development.

[70] Li S., Lu J., Chen Y., Xiong N., Li L., Zhang J., Yang H., Wu C., Zeng H., Liu Y. (2017). MCP-1-induced ERK/GSK-3B/Snail signaling facilitates the epithelial-mesenchymal transition and promotes the migration of MCF-7 human breast carcinoma cells. Cell Mol. Immunol..

[71] Tsai L.K., Wang Z., Munasinghe J., Leng Y., Leeds P., Chuang D.M. (2011). Mesenchymal stem cells primed with valproate and lithium robustly migrate to infarcted regions and facilitate recovery in a stroke model. Stroke.

[72] Dell’Osso L., Del Grande C., Gesi C., Carmassi C., Musetti L. (2016). A new look at an old drug: Neuroprotective effects and therapeutic potentials of lithium salts. Neuropsychiatr. Dis. Treat..

[73] Wei W., Ao Q., Wang X., Cao Y., Liu Y., Zheng S.G., Tian X. (2021). Mesenchymal stem cell-derived exosomes: A promising biological tool in nanomedicine. Front. Pharmacol..

[74] Gurung S., Perocheau D., Touramanidou L., Baruteau J. (2021). The exosome journey: From biogenesis to uptake and intracellular signalling. Cell Commun. Signal..

[75] Alonso-Alsonso M.L., Garcia-Posadas L., Diebold Y. (2021). Extracellular vesicles from human adipose-derived mesenchymal stem cells: A review of common cargos. Stem Cell Rev. Rep..

[76] Shi J., Zhao Y.C., Niu Z.F., Fan H.J., Hou S.K., Guo X.Q., Sang L., Lv Q. (2021). Mesenchymal stem cell-derived small extracellular vesicles in the treatment of human diseases: Progress and prospect. World J. Stem Cells..

[77] Capomaccio S., Cappelli K., Bazzucchi C., Coletti M., Gialletti R., Moriconi F., Passamonti F., Pepe M., Petrini S., Mecocci S. (2019). Equine adipose-derived mesenchymal stromal cells release extracellular vesicles enclosing different subsets of small RNAs. Stem Cells Int..

[78] Loussouarm C., Pers Y.M., Bony C., Jorgensen C., Noel D. (2021). Mesenchymal stromal cell-derived extracellular vesicles regulate the mitochondrial metabolism via transfer of miRNAs. Front. Immunol..

[79] Ramos T.L., Sanchez-Abarca L.I., Muntion S., Preciado S., Puig N., Lopez-Ruano G., Hernandez-Hernandez A., Redondo A., Ortega R., Rodriguez C. (2016). MSC surface markers (CD44, CD73, CD90) can identify human MSC-derived extracellular vesicles by conventional flow cytometry. Cell Commun. Signal..

[80] Sung D.K., Chang Y.S., Sung S.I., Ahn S.Y., Park W.S. (2019). Thrombin preconditioning of extracellular vesicles derived from mesenchymal stem cells accelerates cutaneous wound healing by boosting their biogenesis and enriching cargo content. J. Clin. Med..

[81] Sheike G.V., Yin Y., Jang S.C., Lassar C., Wennmalm S., Hoffman H.J., Li L., Gho Y.S., Nilsson J.A., Lotvall J. (2019). Endosomal signaling via exosome surface TGFbeta-1. J. Extracell. Vesicles.

[82] Sun W., Luo J.D., Jiang H., Duan D.D. (2018). Tumor exosomes: A double-edged sword in cancer therapy. Acta Pharmacol. Sin..

[83] Yamanaka S. (2012). Induced pluripotent stem cells: Past, present and future. Cell Stem Cell.

[84] Skalova S., Svadlakova T., Qureshi W.M.S., Dev K., Mokry J. (2015). Induced pluripotent stem cells and their use in cardiac and neural regenerative medicine. Int. J. Mol. Sci..

[85] James E.C., Tomaskovic-Crook E., Crook J.M. (2021). Bioengineering clinically relevant cardiomyocytes and cardiac tissues from pluripotent stem cells. Int. J. Mol. Sci..

[86] Ford E., Pearlman J., Ruan T., Manion J., Waller M., Neely G.G., Caron L. (2020). Human pluripotent stem cells-based therapies for neurodegenerative diseases: Current status and challenges. Cells.

[87] Guo X., Tang L., Tang X. (2021). Current developments in cell replacement therapy for Parkinson’s disease. Neuroscience.

[88] Anderson N.C., Chen P.F., Meganathan K., Saber W.A., Petersen A.J., Bhattacharyya A., Kroll K.L., Sahin M., Cross-IDDRC Human Stem Cell Working Group (2021). Balancing serendipity and reproducibility: Pluripotent stem cells as experimental systems for intellectual and developmental disorders. Stem Cell Rep..

[89] Antonov S.A., Novosadova E.V. (2021). Current state-of-the-art and unresolved problems in using human pluripotent stem cell-derived dopamine neurons for Parkinson’s disease drug development. Int. J. Mol. Sci..

[90] Croft A.S., Illien-Junger S., Grad S., Guerrero J., Wangler S., Gantenbein B. (2021). The application of mesenchymal stem cells and their homing capabilities to regenerate the intervertebral disc. Int J. Mol. Sci..

[91] Thulabandu V., Chen D., Atit R.P. (2018). Dermal fibroblast in cutaneous development and healing. Wiley Interdiscip. Rev. WIREs Dev. Biol..

[92] Griffin M.F., DesJardins-Park H.E., Mascharak S., Borreli M.R., Longaker M.T. (2020). Understanding the impact of fibroblast heterogeneity on skin fibrosis. Dis. Model. Mech..

[93] Shaw T.J., Rognoni E. (2020). Dissecting fibroblast heterogeneity in health and fibrotic disease. Curr. Rheumatol. Rep..

[94] Hicks D.A., Jones A.C., Corbett N.J., Fisher K., Pickering-Brown S.M., Ashe M.P., Hooper N.M. (2020). Extracellular vesicles isolated from human induced pluripotent stem cell-derived neurons contain transcriptional network. Neurochem. Res..

[95] Hirabayashi J., Tateno H., Onuma Y., Ito Y. (2015). A novel probe as surface glycan marker of pluripotent stem cells: Research outcomes and application to regenerative medicine. Adv. Healthc. Mater..

[96] Hart D.A., Kydd A.S., Frank C.B., Hildebrand K.A. (2004). Tissue repair in rheumatoid arthritis: Challenges and opportunities in the face of a systemic inflammatory disease. Best Pract. Res. Clin. Rheumatol..

[97] Liu L., Chen J.X., Zhang X.W., Sun Q., Yang L., Liu A., Hu S., Guo F., Liu S., Huang Y. (2018). Chemokine receptor 7 overexpression promotes mesenchymal stem cell migration and proliferation via secreting chemokine ligand 12. Sci. Rep..

[98] Lee D.Y., Cha B.H., Jung M., Kim A.S., Bull D.A., Won D.Y. (2018). Cell surface engineering and application in cell delivery to heart diseases. J. Biol. Eng..

[99] Lee J., Dykstra B., Spenser J.A., Kenny L.L., Greiner D.L., Shultz L.D., Brehm M.A., Lin C.P., Sackstein R., Rossi D.J. (2017). mRNA-mediated glycoengineering ameliorates deficient homing of human stem cell-derived hematopoietic progenitors. J. Clin. Investig..

[100] Narimatsu Y., Bull C., Chen Y.H., Wandall H.H., Yang Z. (2021). Genetic glycoengineering in mammalian cells. J. Biol. Chem..

[101] Du J., Meledeo M.A., Wang Z., Khanna H.S., Panchuri V.D.P., Yarema K.J. (2009). Metabolic glycoengineering: Sialic acid and beyond. Glycobiology.

[102] Du J., Yarema K.J. (2010). Carbohydrate engineered cells for regenerative medicine. Adv. Drug Deliv. Rev..

[103] Cheng Z., Ou L., Zhou X., Li F., Jia X., Zhang Y., Liu X., Li Y., Ward C.A., Melo L.G. (2008). Targeted migration of mesenchymal stem cells modified with CXCR4 gene to infarcted myocardium improves cardiac performance. Mol. Ther..

[104] Dykstra B., Lee J., Mortensen L.J., Yu H., Wu Z.L., Lin C.P., Rossi D.J., Sackstein R. (2016). Glycoengineeering of E-selectin ligands by intracellular versus extracellular fucosylation differentially affects osteotropism of human mesenchymal stem cells. Stem Cells.

[105] Lo C.Y., Weil B.R., Palka B.A., Momeni A., Canty J.M., Neelamegham S. (2016). Cell surface glycoengineering improves selectin-mediated adhesion of mesenchymal stem cells (MSCs) and cardiosphere-derived cells (CDCs): Pilot validation in porcine ischemia-reperfusion model. Biomaterials.

[106] Wei Z., Chen Z., Zhao Y., Fan F., Xiong W., Song S., Yin Y., Hu J., Yang K., Yang L. (2021). Mononuclear phagocyte system blockade using extracellular vesicles modified with CD47 on membrane surface for myocardial infarction reperfusion injury treatment. Biomaterials.

[107] Kuang S., He F., Liu G., Sun X., Dai J., Chi A., Tang Y., Li Z., Gao Y., Deng C. (2021). CCR2-engineered mesenchymal stromal cells accelerate diabetic wound healing by restoring immunological homeostasis. Biomaterials.

[108] Liao N., Zhang D., Wu M., Yang H., Liu X., Song L. (2021). Enhancing therapeutic effects and in vivo tracking of adipose tissue-derived mesenchymal stem cells for liver injury using bioorthagonal click chemistry. Nanoscale.

[109] Yan H., Mi X., Midgley A.C., Du X., Huang Z., Wei T., Liu R., Ma T., Zhi D., Zhu D. (2020). Targeted repair of vascular injury by adipose-derived stem cells modified with P-selectin binding peptide. Adv. Sci..

